# *Nucleoporin98-96* Function Is Required for Transit Amplification Divisions in the Germ Line of *Drosophila melanogaster*

**DOI:** 10.1371/journal.pone.0025087

**Published:** 2011-09-16

**Authors:** Benjamin B. Parrott, Yuting Chiang, Alicia Hudson, Angshuman Sarkar, Antoine Guichet, Cordula Schulz

**Affiliations:** 1 Department of Cellular Biology, University of Georgia, Athens, Georgia, United States of America; 2 Department of Psychology, Harvard University, Cambridge, Massachusetts, United States of America; 3 Biological Science, BITS Pilani Goa Campus, Zuarinagar, India; 4 Equipe Polarité et Morphogenèse, Institut Jacques Monod, Paris, France; University of Texas MD Anderson Cancer Center, United States of America

## Abstract

Production of specialized cells from precursors depends on a tightly regulated sequence of proliferation and differentiation steps. In the gonad of *Drosophila melanogaster*, the daughters of germ line stem cells (GSC) go through precisely four rounds of transit amplification divisions to produce clusters of 16 interconnected germ line cells before entering a stereotypic differentiation cascade. Here we show that animals harbouring a transposon insertion in the center of the complex *nucleoporin98-96* (*nup98-96*) locus had severe defects in the early steps of this developmental program, ultimately leading to germ cell loss and sterility. A phenotypic analysis indicated that flies carrying the transposon insertion, designated *nup98-96^2288^*, had dramatically reduced numbers of germ line cells. In contrast to controls, mutant testes contained many solitary germ line cells that had committed to differentiation as well as abnormally small clusters of two, four or eight differentiating germ line cells. This indicates that mutant GSCs rather differentiated than self-renewed, and that these GSCs and their daughters initiated the differentiation cascade after zero, or less than four rounds of amplification divisions. This phenotype remained unaffected by hyper-activation of signalling pathways that normally result in excessive proliferation of GSCs and their daughters. Expression of wildtype *nup98-96* specifically in the germ line cells of mutant animals fully restored development of the GSC lineage, demonstrating that the effect of the mutation is cell-autonomous. Nucleoporins are the structural components of the nucleopore and have also been implicated in transcriptional regulation of specific target genes. The nuclear envelopes of germ cells and general nucleocytoplasmic transport in *nup98-96* mutant animals appeared normal, leading us to propose that *Drosophila nup98-96* mediates the transport or transcription of targets required for the developmental timing between amplification and differentiation.

## Introduction

In development and tissue homeostasis, the proliferation of precursor cells and the initiation of terminal differentiation are temporally separated. For example, regeneration of organs typically involves proliferation of de-differentiated or pre-existing pluripotent cells followed by coordinated differentiation. Tissue homeostasis from self-renewing populations of stem cells follows a similar two-step process. First, stem cell daughters exiting the stem cell fate multiply by transit amplification divisions to create a pool of precursor cells. Then these precursors develop into specialized cell types through a precisely coordinated cascade of differentiation events [1], [2]. The *Drosophila* gonad has served as a highly successful model for elucidating many of the signaling pathways that regulate the cell fate, amplification, and differentiation of the GSC lineage [3], [4]. However, comparatively little is known about the molecules and mechanisms that coordinate developmental timing and, specifically, the timing between amplification and differentiation of stem cell daughters.

Here, we show that a normal balance between transit amplification divisions and terminal differentiation depends on the complex *nucleoporin98-96* (*nup98-96*) locus. Nucleoporins are structural components of the nuclear pore and have well-established functions in nucleo-cytoplasmic transport as well as the breakdown and re-assembly of the nuclear envelope during mitosis [5]–[7]. More recently, it has become clear that members of this protein family also contribute to the regulation of developmental processes via their effect on gene transcription. Specifically, *Drosophila* Nup98 was found to associate with actively transcribed chromatin in salivary glands of 3^rd^ instar wildtype larvae in a manner dependent on Ecdysone, a steroid hormone and key regulator of molting and metamorphosis. Transcriptional up-regulation in response to Ecdysone is correlated with increased chromatin occupancy of Nup98 while down-regulation correlated with a decrease in Nup98 chromatin binding. Transcriptional profiling of *Drosophila* S2 cells further established that Nup98 and a second nuleoporin, Sec13, control the transcription of specific target genes regulating developmental transitions and the cell cycle [8], [9].

The highly conserved *nup98-96* locus is complex and gives rise to two distinct proteins, Nup98 and Nup96. Alternative splicing generates two transcripts in *Drosophila*: a short mRNA containing an open reading frame for only Nup98, and a long mRNA with an open reading frame for a Nup98-Nup96 poly-protein. Processing by autocatalytic cleavage subsequently separates the two functional units, Nup98 and Nup96. In *Drosophila*, *nup98-96* transcripts were detected at all stages of development [10]–[12]. Mutations harbouring a stop codon in Nup98, and thus presumably eliminating both Nup98 and Nup96 function, are associated with lethality prior to metamorphosis, possibly reflecting the role of Nup98 in Ecdyson-dependent gene transcription [12].

Here, we investigate the role of the *nup98-96* locus in the germ line stem cell lineage. In a screen for mutations effecting the development of germ line cells, we identified a transposon-insertion in the center of the *nup98-96* locus. In *Drosophila* wildtype males, the daughters of GSCs amplify by exactly four rounds of mitosis with incomplete cytokinesis to produce clusters of 16 spermatogonia that remain interconnected by cytoplasmic bridges. After mitosis, the spermatogonia become spermatocytes, which enter the terminal differentiation cascade. The first step of terminal differentiation is an extreme increase in germ cell size accompanied by the expression of most of the genes that mediate subsequent differentiation steps. Subsequently, the spermatocytes undergo meiosis and develop into spermatids [13], [14]. In the gonads of males homozygous for the *nup98-96^2288^* mutation, or harbouring *nup98-96^2288^* in trans to a deficiency that uncovers the locus (*Df(3R)mbc-R1*), GSCs and their daughters appeared to differentiate into spermatocytes either directly or after less than four rounds of transit amplification divisions. These defects were fully complemented by expression of *nup98-96* specifically in the germ line, revealing a cell autonomous mode of action. Manipulations of signalling pathways that result in the over-proliferation of germ line cells in otherwise wildtype testes did not attenuate the *nup98-96^2288^* phenotype. As the nuclear pore of mutant animals showed no obvious defects, we propose that the defects in *nup98-96^2288^*/*Df(3R)mbc-R1* mutant animals are due to the lack of either nucleocytoplasmic transport or transcription of as yet unidentified factors required for timing the transition between amplification and terminal differentiation.

## Results

### The nup98-96 locus is required for maintaining germ line cells in an undifferentiated state

Animals carrying the *nup98-96^2288^* mutation were first identified in a genetic screen for sterile animals with abnormally small gonads. We subsequently observed the same gonad phenotype in animals trans-heterozygous for *nup98-96^2288^* and *Df(3R)mbc-R1*, suggesting that the *nup98-96^2288^* allele acts as a strong allele with respect to the gonad phenotype. No other morphological abnormalities were obvious in these animals, implying that *nup98-96^2288^* is a mutation with a specific effect on gametogenesis.

**Figure 1:**
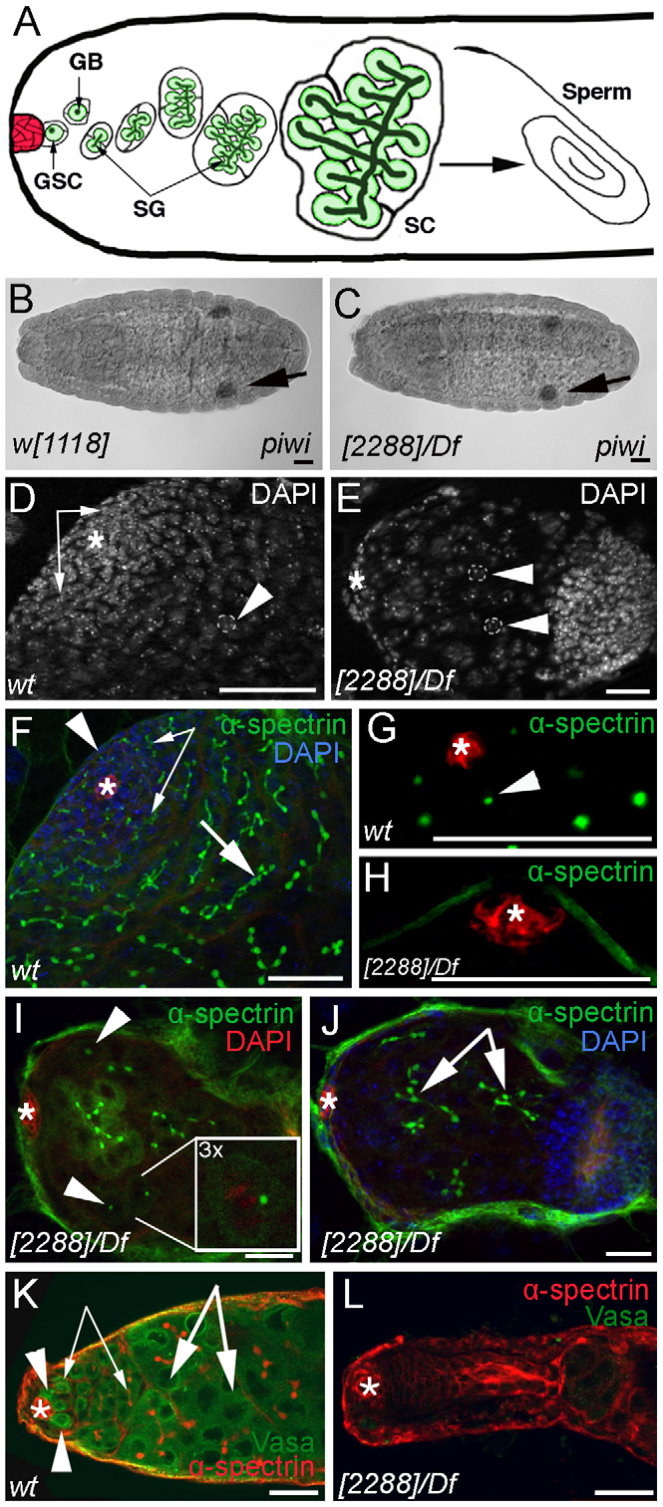
Germ line cells are not maintained at early stages and differentiate in *nup98-96^2288^/Df(3R)mbc-R1* mutant testes. (A) Drawing showing the stages of germ line cell differentiation in testes. GSC: germ line stem cell, GB: gonialblast, SG: spermatogonia, SC: spermatocytes. Black circles: somatic support cells enclosing the germ line cells. (B, C) *In situ* hybridization with a *piwi*-RNA-probe to (B) *w^1118^* and (C) *nup98-96^2288^/Df(3R)mbc-R1* mutant embryos. Anterior to the left. (D–J) 3^rd^ instar larval testes. (D, E) DNA in D) the apical region of a wildtype (wt), and (E) a whole *nup98-96^2288^/Df(3R)mbc-R1* testis. Arrows point to early stage germ line cell nuclei, arrowheads point to spermatocyte nuclei that are in addition outlined by grey dotted circles. The small, strong DAPI-positive signals at the posterior (right) end of the testes correspond to the nuclei of somatic precursor cells that will develop into the most basal somatic structures of the testes during pupal stage. (F–L) FasIII and asterisks label the hub. Arrowheads point to spectrosomes, small arrow points to the small, branched fusomes as normally seen in the spermatogonia, large arrows point to the wide, long, branched fusomes as normally seen in the spermatocytes. (F) Apical region of a wildtype testis. (G, H) High magnification of apical tips of testes from (G) wildtype and (H) *nup98-96^2288^/Df(3R)mbc-R1.* (I, J) Whole *nup98-96^2288^/Df(3R)mbc-R1* testes. Note the inset in (I) showing the spectrosome (green) and DNA (red) in a single, large germ line cell. (K, L) Apical regions of adult testes from (K) wildtype and (L) *nup98-96^2288^/Df(3R)mbc-R1.* Scale bars: 30 µm.

In testes from *nup98-96^2288^*/*Df(3R)mbc-R1* mutant animals (hence forth referred to as *nup98-96^2288^*/*Df(3R)mbc-R1* testes), the germ line cells were progressively lost with increasing age of the animal. Normally, the germ line cells are arranged in a spatio-temporal gradient along the apical to basal axis of the testis (Figure 1A). GSCs are confined to the apical tip and surround a group of somatic cells, called the hub (red in Figure 1A). Their immediate daughters (gonialblasts) and clusters of between two and 16 interconnected cells in the process of transit amplification divisions (spermatogonia) become displaced basally and are found a short distance from the hub. Large spermatocytes that have initiated the differentiation cascade and mature spermatids occupy more basal positions within the testis. At all stages of development, GSCs and their progeny are fully enclosed by somatic support cells (black circles in Figure 1A). This germ cell microenvironment, or niche, provides external cues that regulate stem cell self-renewal, stem cell daughter amplification, and germ line differentiation [15], [16].

*In situ* hybridization with a gonad-specific probe (*piwi*-RNA) revealed that the gonads from *nup98-96^2288^/Df(3R)mbc-R1* mutant embryos were similar in size to gonads from control animals (in the following referred to as control testes, compare Figure 1C to 1B, n>50). However, by the 3^rd^ instar larval stage, *nup98-96^2288^/Df(3R)mbc-R1* testes were noticeably smaller than control testes (compare Figure 1E to 1D, n>100), and did not contain any early stage germ line cells (GSCs, gonialblasts, or spermatogonia). In preparations stained with 4′,6-diamidino-2-phenylindole (DAPI), the nuclei of early stage germ line cells appear as characteristic small bright signals (Figure 1D, arrows) due to the small size of their nuclei, whereas spermatocytes that have initiated the differentiation cascade have larger, less brightly staining nuclei (Figure 1D, arrowhead). In contrast to control testes, the apical region of *nup98-96^2288^/Df(3R)mbc-R1* testes did not contain many small, brightly stained nuclei, suggesting that early stage germ line cells were depleted. However, larger and less brightly staining nuclei characteristic of spermatocytes were present in *nup98-96^2288^/Df(3R)mbc-R1* testes (Figure 1E, arrowheads).

Labelling with an antibody against α-spectrin confirmed that 3^rd^ instar larval *nup98-96^2288^/Df(3R)mbc-R1* testes lacked transit-amplifying spermatogonia, but contained germ line cells at the spermatocyte stage. In germ line cells, α-spectrin labels a sub-cellular structure called the fusome, and the shape and the size of fusomes is indicative of the germ line cell's developmental stage [17]. The GSCs and the gonialblasts contain round fusomes, commonly referred to as spectrosomes. The spectrosomes containing GSCs (Figure 1F, G arrowheads) are found next to the hub (red and marked with an asterisk in Figure 1F–1L). Clusters of interconnected germ line cells contain branched fusomes that reach through their intercellular bridges. α-spectrin-staining reveals that spermatogonia in transit amplifying divisions have small, branched fusomes and are located relatively close to the hub while the spermatocytes have large, branched fusomes and are found more basally (Figure 1F, small and large arrows, respectively).

3^rd^ instar *nup98-96^2288^/Df(3R)mbc-R1* testes did not contain cells with α-spectrin-positive spectrosomes located next to the hub (Figure 1H, n>50). Cells with α-spectrin-positive structures were mostly found toward the middle and basal region of mutant testes (Figure 1I). Germ line cells with round spectrosomes characteristic of GSCs were detected. However, these cells were larger than GSCs (Figure 1I, arrowheads) and contained large and less brightly DAPI-stained nuclei typical of spermatocytes (Figure 1I, inset). We propose that these solitary differentiating germ line cells originated from GSCs and gonialblasts that failed to undergo amplification divisions. In addition, we detected many wide fusomes (Figure 1J, arrows) that connected large germ line cells with large nuclei and less brightly staining DNA (compare Figure 1J–1E), similar to wildtype spermatocytes. In contrast to the fusomes in 16 cell stage spermatocytes in control testes, the fusomes in the *nup98-96^2288^/Df(3R)mbc-R1* testes consistently had fewer than 16 branches and appeared to connect only two, four or eight spermatocytes. This implies that most of the germ line cells of *nup98-96^2288^/Df(3R)mbc-R1* testes only went through one, two or three instead of the stereotypical four rounds of amplification divisions. In support of this conclusion, the *nup98-96^2288^/Df(3R)mbc-R1* testes contained far fewer germ line cells than control testes: on average, only 34 α-spectrin-positive cells were found per mutant testis (s.d. 20, range: 0–54, n = 40) compared to several hundred α-spectrin-positive cells found in control testes (n>50).

Adult *nup98-96^2288^/Df(3R)mbc-R1* testes completely lacked early stage germ line cells and, on the basis of immuno-labelling with the germ line cell markers Vasa and α-spectrin, rarely contained late stage germ line cells. In control testes, Vasa-positive GSCs (arrowheads in Figure 1K) form a rosette around the apical hub (n>100). Vasa-positive spermatogonia are found at a distance from the tip in the apical region of the testes (Figure 1K, small arrows), whereas large Vasa-positive spermatocytes are located more toward the base (Figure 1K, large arrows). Double staining with Vasa- and α-spectrin-antisera revealed that 98% of adult *nup98-96^2288^/Df(3R)mbc-R1* testes did not contain any germ line cells (Figure 1L, n>100). The remaining 2% of the mutant testes contained either two or four large, Vasa-positive spermatocytes located in the testis coil, or a few immature sperm bundles (data not shown). We conclude that the failure to undergo the normal numbers of amplification divisions completely exhausted the germ line of *nup98-96^2288^/Df(3R)mbc-R1* males.

### *nup98-96^2288^* plays a parallel role in the female gonad

**Figure 2:**
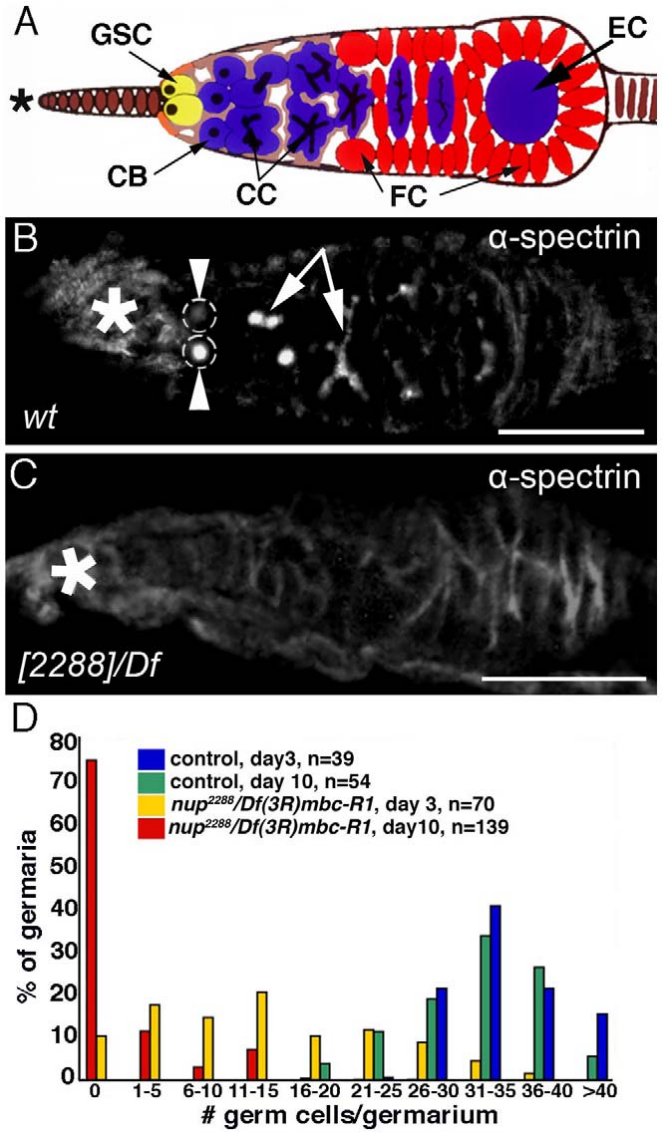
Germ line cell loss in *nup98-96^2288^/Df(3R)mbc-R1* mutant germaria. (A) Drawing showing the organization of GSCs and their daughters in a wildtype germarium. GSC: germ line stem cell, CB: cystoblast, CC: cystocytes, EC: egg chamber, FC: follicle cells, black marking in germ line cells: spectrosomes and branched fusomes. (B, C) Germaria. Asterisks mark the apical tips, arrowheads point to spectrosomes in the GSCs (grey dotted circles), arrows point to branched fusomes. Note that only a section of the fusomes is in focal plane. Scale bars: 50 µm. (D) Histogram depicting the number of Vasa-positive germ line cells in the germaria at different time points post-eclosion. Genotypes are as indicated.

Much like the testis, the gonad of female flies is organized in an apical-to-basal differentiation gradient of germ line cells. GSCs lie at the apical tip of the germarium. The stem cell daughters (cystoblasts), and their transit amplifying progeny (cystocytes) become progressively displaced away from the tip toward the base (Figure 2A) [18]. GSCs and cystoblasts are characterized by the presence of a α-spectrin-positive spectrosome (Figure 2B, arrowheads) whereas the interconnected cystocytes contain branched fusomes (Figure 2B, arrows). Labelling with α-spectrin antibodies revealed the absence of both spectrosomes and fusomes in most germaria from *nup98-96^2288^* homozygous (data not shown) and *nup98-96^2288^/Df(3R)mbc-R1* mutant females (Figure 2C, 70%, n>100).

We next investigated if the loss of early stage germ line cells observed in *nup98-96^2288^/Df(3R)mbc-R1* germaria was age-dependent. Ovaries from wildtype control and *nup98-96^2288^/Df(3R)mbc-R1* mutant flies were collected at three and ten days post-eclosion, immuno-labelled with the germ line marker anti-Vasa, and the number of Vasa-positive germ line cells in each germarium was quantified (Figure 2D). We found that the number of germ line cells in control animals did not change significantly between three and ten days post-eclosion. Control germaria contained on average 35 Vasa-positive cells three days post-eclosion (n = 100; standard deviation: 16, range: 21 - >40; blue bars in Figure 2D) and 32 Vasa-positive cells 10 days post-eclosion (n = 100; standard deviation: 16; range: 16 - >40; green bars in Figure 2D). Control germaria without Vasa-positive cells were never observed. In contrast, *nup98-96^2288^/Df(3R)mbc-R1* germaria showed a dramatic loss of germ line cells. Three days post-eclosion, mutant germaria contained an average of 13 Vasa positive cells (n = 100; standard deviation: 27), but this number varied widely (from 0 to 40; yellow bars in Figure 2D). Ten days post-eclosion, mutant germaria, on average, contained only 2 Vasa-positive cells (n = 100; standard deviation: 13; range: 0–15; red bars in Figure 2D). Notably, 75% of the *nup98-96^2288^/Df(3R)mbc-R1* germaria had no detectable germ line cells. We conclude that the *nup98-96^2288^* mutation has similar effects on both male and female early stage germ line cells: in both sexes, the GSC lineage is rapidly depleted with increasing age, presumably due to differentiation.

### The defects in the *nup98-96^2288^/Df(3R)mbc-R1* mutant gonads are due to disruption of a nuclear pore locus

**Figure 3:**
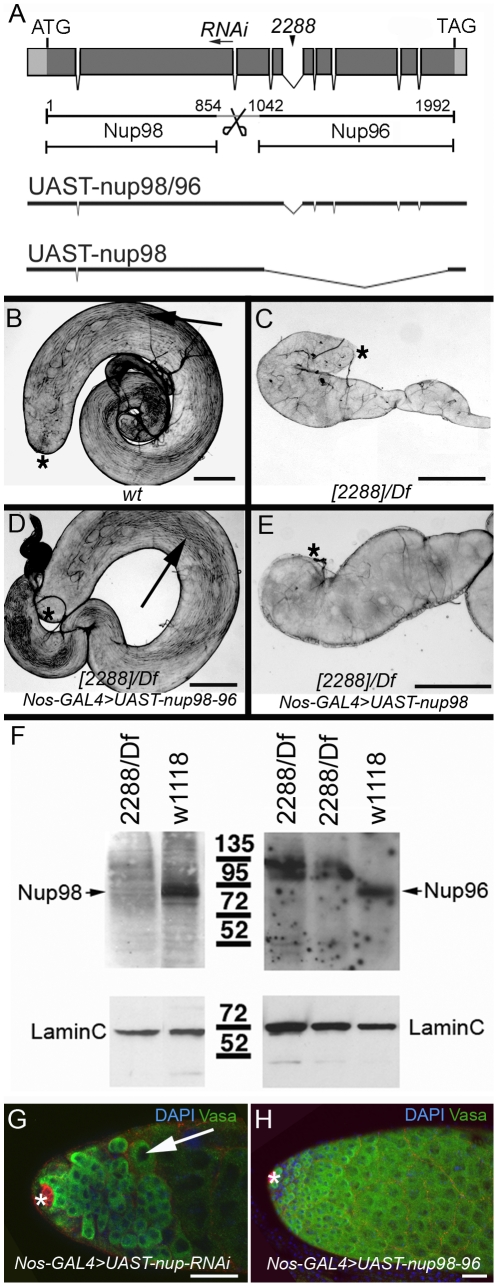
Mutations in *nup98-96* disrupt gametogenesis. (A) Top: Intron-exon structure of the *nup98-96* locus. Coding region in dark grey. Gene products as indicated. Bottom: Rescue constructs containing the whole transcription unit for *nup98-96* or *nup98* only. Arrowhead: Pogo-insertion in *nup98-96^2288^*, arrow: RNAi-sequences. (B-E) Bright field images of whole testes. Genotypes as indicated. Arrows point to sperm. Asterisks mark the apical tips. Scale bars: 100 µm. (F) Western blot analysis. Genotypes and antibodies as indicated. The Nup96 antibody does not detect a 95 kd protein in extracts from mutant animals. Instead, the Nup96 antibody detects a high molecular weight bands that may be abnormal Nup96 protein or Nup98-Nup96 polyprotein. The Nup98 antibody detects extremely low levels a 95 kd protein in the mutant compared to the control. (G, H) Apical tips of adult testes with germ line expression of (G) UAST-*nup98-96*-RNAi (arrow points to a single spermatocyte), and (H) UAST-*nup98-96*. Asterisks mark the apical tips. Scale bars: 30 µm.

We mapped the *nup98-96^2288^* mutation to chromosomal region 95A5 to C10. Complementation tests using mutations in genes along this region revealed that the defects in the mutant animals were due to a disruption of the *nup98-96* locus. Sequencing of genomic DNA from *nup98-96^2288^* mutant animals revealed two changes to the published gene sequence of the *nup98-96* locus. Mutant animals harboured a point mutation that results in an amino acid exchange of the Nup98 coding sequence (CAA to CGA, Glutamine^860^ to Arginine). However, the same amino acid exchange is found in *nup98-96* alleles of *Drosophila pseudoobscura* [19], strongly suggesting it is a natural variant and does not cause the defects associated with the *nup98-96^2288^* allele. In addition, mutant animals carried a *Pogo*-element insertion in the fourth intron of the *nup98-96* locus (Figure 3A, indicated as 2288). This insertion is predicted to disrupt the splicing of exon 4 to exon 5 (encoding the N-terminal portion of Nup96) and thus should specifically prevent the formation of Nup96.

Expression of a rescue construct in the gonads of mutant animals confirmed that the *nup98-96^2288^* mutant phenotype was due to lesions in the *nup98-96* locus. We generated flies carrying cDNAs encoding the two naturally occurring mRNAs under control of Yeast Upstream Activating Sequence. Flies carrying a full-length cDNA encoding *nup98* and *nup96* (UAST-*nup98-96*, Figure 3A) were crossed to flies carrying *gal4*-transactivators to induce tissue specific expression [20]. Expression of UAST-*nup98-96* in germ line cells of *nup98-96^2288^/Df(3R)mbc-R1* mutant males using the germ cell specific driver *nanos-gal4-VP16* (*nos-gal4*) [21] restored spermatogenesis.

An adult wildtype testis is a coiled, tubular organ that is, on average, 2 mm long (n>100) and contains germ line cells at all stages of spermatogenesis, including sperm bundles (Figure 3B, arrow). Adult *nup98-96^2288^*/*Df(3R)mbc-R1* testes were much shorter (Figure 3C) than control testes, measuring only 100–500 µm in length (n>100). In addition, mutant testes contained very few, if any, germ line cells (see above). *nup98-96^2288^*/*Df(3R)mbc-R1* testes with germ line specific expression of UAST-*nup98-96* were of normal size and contained germ line cells at all stages of spermatogenesis, including mature sperm (Figure 3D, arrow, n>100). Expression of the UAST-*nup98-96* construct in the somatic cells of the gonad did not restore spermatogenesis (n>50, data not shown), demonstrating that the defects were due specifically to loss of *nup98-96* from the germ line cells.

Expression of a cDNA (UAST-*nup98*) which only encoded *nup98* (Figure 3A) within the germ line cells from *nup98-96^2288^*/*Df(3R)mbc-R1* testes did not restore spermatogenesis and testes remained small (Figure 3E, n>50). Western blot analysis using protein extracts from whole control and mutant 1^st^ instar larvae revealed that both antibodies (raised against either Nup98 or Nup96) failed to detect significant levels of either protein in the mutant animals (Figure 3F). We conclude that the defects in the *nup98-96^2288^*/*Df(3R)mbc-R1* gonads are due to a strong reduction in both proteins, Nup98 and Nup96.

Confirming the role of the *nup98-96* locus in the GSC lineage, expression of two independent RNA-interference lines targeted against *nup98-96* (Figure 3A, indicated as RNAi) in the germ line cells of otherwise wildtype animals also resulted in progressive loss of early stage germ line cells and the appearance of single cell spermatocytes (Figure 3G, arrow, n>50). Conversely, expression of UAST-*nup98* or UAST-*nup98-96* (Figure 3H) in germ line cells of otherwise wildtype animals did not cause any defects in spermatogenesis (n>50) suggesting that the *nup98-96* locus plays a permissive role in germ line development.

### In *nup98-96^2288^/Df(3R)mbc-R1* animals, the nuclear envelope and general nucleocytoplasmic transport appear normal

**Figure 4:**
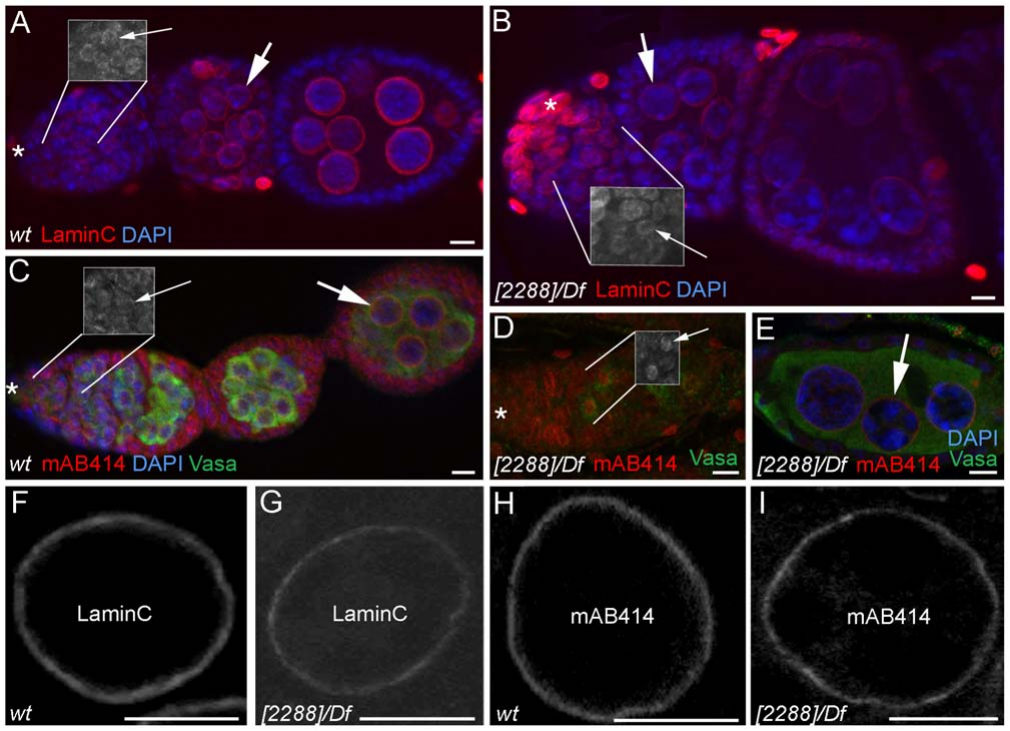
Germ cells from *nup98-96^2288^/Df(3R)mbc-R1* animals have normal nuclear envelopes. (A–E) Images showing nuclear localization around nuclei of early stage germ line cells (see insets) and nurse cells. Small arrows in insets point to early stage germ cell nuclei in the germaria and large arrows in images point to nurse cell nuclei in egg chambers. Immunofluorescence-labelling as indicated. (A, B) Apical region of (A) a wildtype and (B) a *nup98-96^2288^/Df(3R)mbc-R1* ovariole. (C) The apical region of a wildtype ovariole. (D) The germarium and (E) an egg chamber of a *nup98-96^2288^/Df(3R)mbc-R1* ovariole. (F–I) Immuno-labelling of single nurse cell nuclei, antibodies and genotypes as indicated. Asterisks: apical tips of germaria, scale bars: 20 µm.

The molecular nature of *nup98-96* suggests that the proteins play a structural role in germ line cells. We therefore used the nuclear envelope marker LaminC and the nuclear pore marker mAB414 to determine if *nup98-96^2288^* caused any visible alterations in nuclear envelope morphology. This analysis was performed using ovaries from young wildtype and *nup98-96^2288^/Df(3R)mbc-R1* females since the female germ line cells are larger than male germ line cells and thus enable imaging with better sub-cellular resolution. In *nup98-96^2288^/Df(3R)mbc-R1* gonads, LaminC (Figure 4B, 4G), and mAB414 (Figure 4D, 4E, 4I) localization to the nuclear envelopes and nuclear pores of the germ line cells appeared normal compared to the controls (Figure 4A, 4C, 4F, 4H).

**Figure 5:**
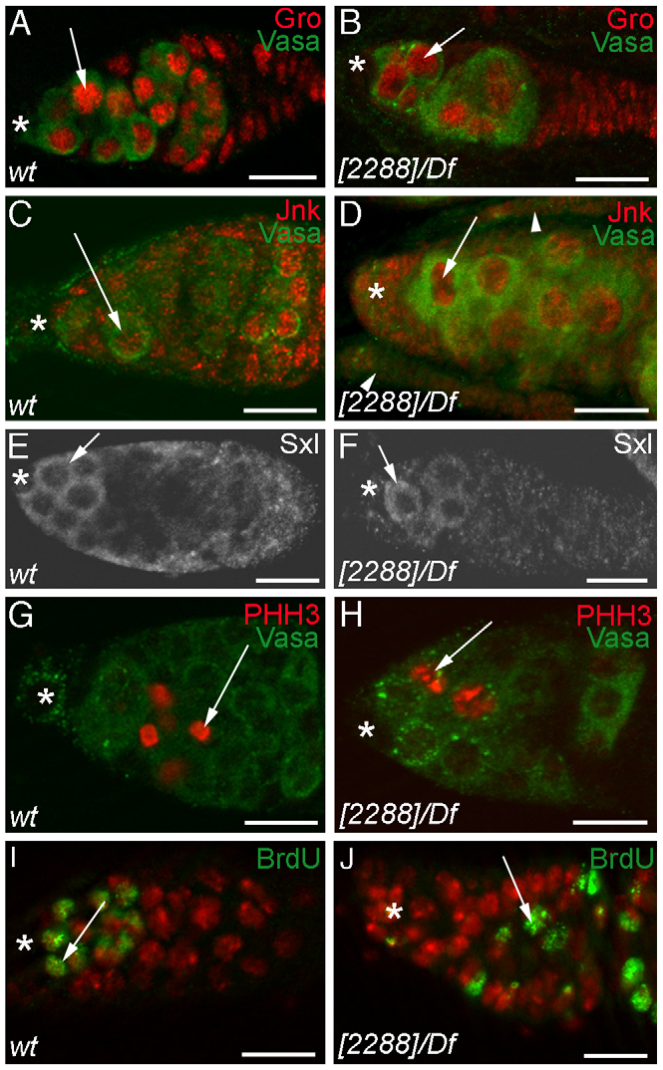
Germ line cells from *nup98-96^2288^/Df(3R)mbc-R1* mutant animals have normal protein localization patterns. Immuno-labelling of germaria, antibodies and genotypes as indicated. (A, B) nuclear Groucho (red) and cytoplasmic Vasa (green); (C, D) nuclear phosphorylated Jun-Kinase (red) and cytoplasmic Vasa (green); note that some germaria (arrowheads) are empty in the *nup98-96^2288^/Df(3R)mbc-R1* mutant ovaries; (E, F) cytoplasmic Sex-lethal in GSCs and gonialblasts; (G, H) nuclear phosphorylated Histone-H3 (red) and cytoplasmic Vasa (green); (I, J) Anti-BRDU (green) and DAPI (red). Asterisks: apical tips, arrows point to intra-cellular protein localizations, scale bars: 50 µm.

Next, we surveyed the effect of the *nup98-96^2288^* mutation on nucleocytoplasmic transport by determining the localization of selected proteins normally found either in the nucleus or in the cytoplasm. The following proteins were assayed: the transcription factors Groucho (Figure 5A, 5B, arrows) and phosphorylated Jun-kinase (Figure 5C, 5D, arrows), the cytoplasmic proteins Vasa (green in Figure 5A–5D and 5G–5H) and Sex-lethal (Figure 5E, 5F, arrows), and the nuclear protein phosphorylated Histone-H3 (Figure 5G, 5H, arrows). As in the controls, Sex-lethal and Vasa were localized to the cytoplasm of *nup98-96^2288^/Df(3R)mbc-R1* germ line cells, indicating that general mRNA export was not disrupted. Likewise, Groucho, phosphorylated Jun-Kinase, and phosphorylated Histone-H3 were localized to the nuclei of both control and *nup98-96^2288^/Df(3R)mbc-R1* germ line cells, showing that the mutant germ line cells are capable of importing these proteins into their nuclei.

Finally on the basis of phosphorylated Histone-H3 and Bromodeoxyuridine (BRDU) labelling, *nup98-96^2288^/Df(3R)mbc-R1* ovaries did not contain germ line cells that appeared to be blocked in S-phase or M-phase of the cell cycle (compare Figure 5H, 5J to Figure 5G, 5I). Thus, our analysis failed to reveal any support for the view that the *nup98-96^2288^* mutation has a noticeable effect on either the structure of the nuclear pore or its function in the general transport of mRNAs and proteins to and from the nucleus.

### The *nup98-96^2288^* mutation causes differentiation of the germ line cells, even in the presence of proliferation-promoting factors

To further explore the role of *nup98-96* in the germ line cells, we tested the genetic interaction of *nup98-96^2288^* with perturbations in signalling pathways that regulate early stage germ line cells. In wildtype ovaries, somatic cap cells signal via the Transforming Growth Factor-β (TGF-β pathway to the adjacent GSCs to regulate their decision between stem cell and cystoblast fate. Upon receptor activation, the TGF-β signal transducers, Mad and Medea, translocate into the nucleus and silence transcription of the differentiation factor *bag of marbles* (*bam*) in the GSCs. Therefore, Bam is normally not found in the cytoplasm of GSCs but is found in the cytoplasm of cystoblasts and cystocytes. Overexpression of *bam* within the germ line cells of otherwise wildtype ovaries causes the germ line cells to be lost, first from the GSC position and then from the entire germaria [22], [23].

**Figure 6:**
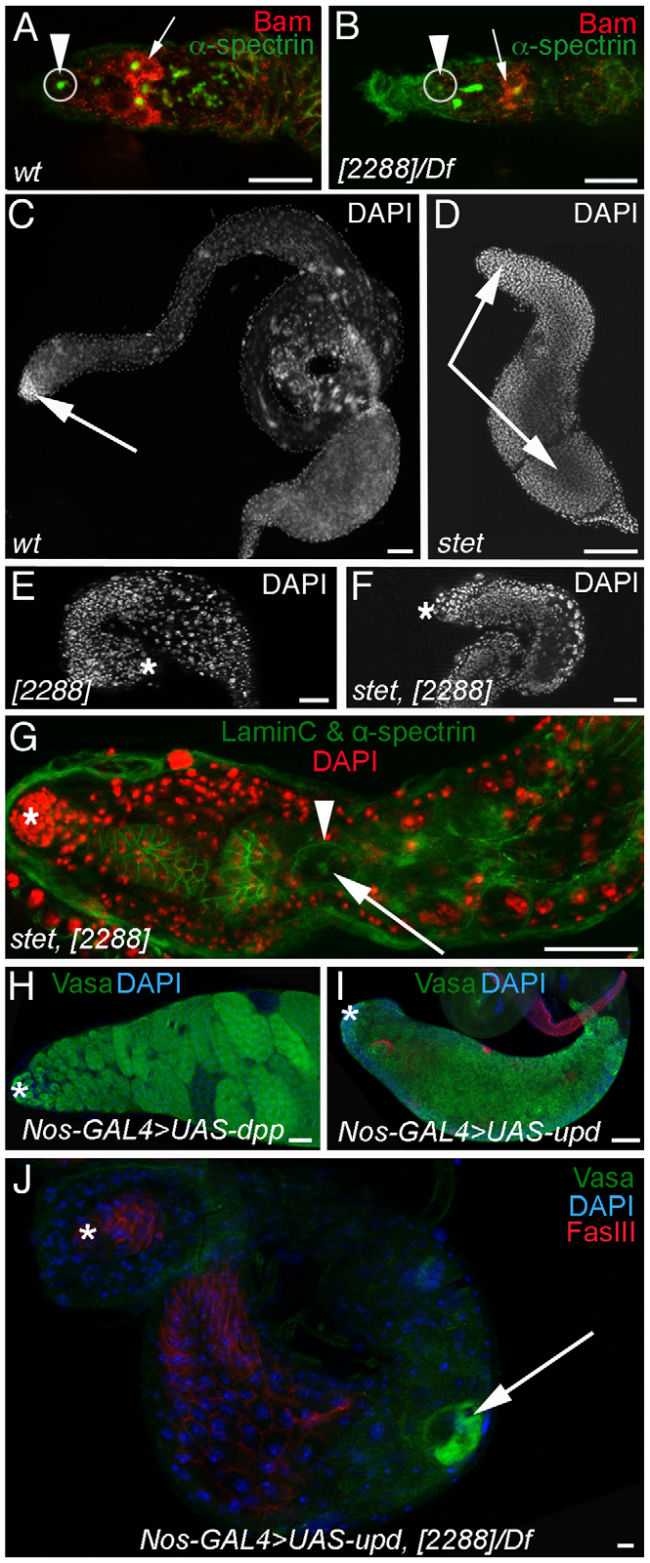
Nup98-96 acts upstream of signalling pathways regulating early stage germ line cells. (A, B) Immuno-labelling of germaria from (A) wildtype and (B) *nup98-96^2288^/Df(3R)mbc-R1* mutant females. Arrowheads point to Bam-negative GSCs (circled in grey), arrows point to Bam-positive cells, scale bars: 50 µm. (C-J) Adult testes, genotypes as indicated. (C–F) DNA-labelling (DAPI), arrows point to early stage germ line cells. (G) Whole *stet^1^; nup98-96^2288^* testis showing a single spermatocyte (arrowhead) with a spectrosome (arrow). (H, I) Hyper-activation of signalling pathways results in accumulation of early stage germ line cells. (H) Apical region of a testis with germ line expression of *dpp*, and (I) whole testis with germ line expression of *upd*. (J) Whole *nup98-96^2288^/Df(3R)mbc-R1* testis with germ line expression of *upd*. Arrow points to single spermatocyte. Asterisks mark the testes tips, scale bars: 30 µm.

The expression pattern of Bam is an excellent tool for determining whether a defect in TGF-β signalling exists in mutant ovaries. In ovaries from freshly eclosed *nup98-96^2288^/Df(3R)mbc-R1* females, Bam was expressed in a pattern similar to that seen in wildtype ovaries. As in wildtype germaria (Figure 6A, arrowhead), the *nup98-96* mutant GSCs located next to the apical tip did not contain cytoplasmic Bam (Figure 6B, arrowhead). In *nup98-96^2288^/Df(3R)mbc-R1* germaria, GSC daughters displaced away from the GSC position showed normal cytoplasmic Bam expression (Figure 6B, arrow), as seen in wildtype germaria (Figure 6A, arrow). We conclude that TGF-β signalling from the soma to the GSCs was not disrupted in *nup98-96^2288^/Df(3R)mbc-R1* mutant germaria.

This conclusion is consistent with the differences between the gonad phenotypes observed in flies with mutations in TGF-β signalling pathway and *nup98-96^2288^/Df(3R)mbc-R1* mutant animals. In both genders, loss of TGF-β signalling specifically causes GSC loss. However, upon loss of TGF-β signalling, the GSC daughters undergo normal numbers of amplification divisions [24], [25]. In contrast, *nup98-96^2288^/Df(3R)mbc-R1* mutant GSCs and their daughters differentiated either directly or after undergoing fewer than the normal four rounds of mitosis.

Loss of signalling via the Epidermal Growth Factor Receptor (EGFR) has an opposite effect on germ line cells than the *nup98-96^2288^* mutation does. EGFR-dependent signalling promotes the growth of the somatic support cells that surround the germ line cells and form their microenvironment (black circles in Figure 1A). Depletion of EGFR signalling, for example via loss of Stet, an enzyme required for processing the EGFR ligand, results in a loss of this regulatory microenvironment. As a consequence, the germ line cells over-proliferate and produce hundreds of early stage germ line cells, which populate the entire testis [26], [27].

We next investigated if germ cells mutant for the *nup98-96^2288^* mutation fail to proliferate in *stet* mutant background by creating double mutant animals. Early stage germ line cells that stain brightly with the nuclear dye DAPI were confined to the tip of wildtype testes (Figure 6C, arrow) but filled the testes of animals homozygous for the strong *stet^1^* allele (Figure 6D, arrows) [24]. In contrast, testes from *nup98-96^2288^* mutant animals were much smaller than *stet^1^*-testes (compare Figure 6E to 6D) and contained few brightly stained, small nuclei (Figure 6E). Likewise, *stet^1^; nup98-96^2288^* double-homozygous animals were smaller than *stet^1^*-testes (compare Figure 6F to 6D) and also contained few brightly stained, small nuclei (Figure 6F). Immuno-labelling with antibodies against α-spectrin revealed few germ line cells in *stet^1^; nup98-96^2288^* double-mutant testes, all of which were large spermatocytes (Figure 6G, arrowhead) and had a spectrosome (Figure 6G, arrow) similar to the spermatocytes in *nup98-96^2288^/Df(3R)mbc-R1* testes. These results indicate that *nup98-96^2288^* can suppress the germ line defects associated with loss of EGFR signalling.

We next determined whether we would see the same effect of *nup98-96^2288^* on overproliferation phenotypes resulting from the hyper-activation of signalling pathways. Hyper-activation of the TGF-β pathway in otherwise wildtype testes forces spermatogonia to proliferate beyond the normal four rounds of amplification division, producing clusters of 64, 128, or more spermatagonia that ultimately die [26], [27]. Hyperactivation of the Janus Kinase/Signal Transducer and Activator of Transcription (JaK/STAT) pathway in otherwise wildtype animals results in testes that are filled with thousands of cells resembling GSCs and gonialblasts [28], [29]. Both phenotypes could be reproduced by either over-expression of the TGF-β ligand *decapentaplegic (dpp)* (Figure 6H, n>50), or the JaK/STAT ligand *unpaired (upd)* (Figure 6I, n>50), in the germ line cells of otherwise wildtype animals. In contrast, over-expression of either ligand in the germ line cells of *nup98-96^2288^* animals failed to increase the number of early stage germ line cells. Instead, the testes were depleted of early stage germ line cells and, occasionally, a spermatocyte was observed (Figure 6J, arrow, n>50). The interaction of *nup98-96^2288^* with EGFR, TGF-β, and JaK/STAT signalling strongly argue that *nup98-96* function is an essential prerequisite for maintaining germ line cells in an undifferentiated state.

## Discussion

The *nup98-96^2288^* mutation disrupts the normal progression of germ line cells through gametogenesis in both male and female flies. Zero or very few germ line cells were found in adult animals. A developmental analysis revealed that the loss of early stage germ line cells was due to differentiation of GSCs. *nup98-96^2288^/Df(3R)mbc-R1* testes contained only late stage germ line cells that were similar to wildtype spermatocytes. Spermatocytes normally develop in clusters of 16 cells that are derived from a single gonialblast undergoing four rounds of transit amplification divisions with incomplete cytokinesis. In contrast, the late stage germ line cells of *nup98-96^2288^/Df(3R)mbc-R1* testes were either solitary, with a single large spectrosome, or part of small clusters of cells with wide, branched fusomes that connected only two to eight cells. This finding implies that GSCs and gonialblasts initiated the spermatocyte differentiation cascade either without or after a reduced number of transit amplification divisions.

The *nup98-96* gene products are structural components of the nuclear pores and it seems possible that the defects seen in the *nup98-96^2288^/Df(3R)mbc-R1* gonads may have been caused by generic defects in the nuclear pores or nuclear envelope. However, the nuclear envelopes of *nup98-96^2288^* germ line cells did not exhibit defects apparent by immuno-fluorescence experiments, and the localization of several nuclear markers as well as cytoplasmic markers was unaffected in *nup98-96^2288^/Df(3R)mbc-R1* gonads. Furthermore, a reduction in the numbers of amplification divisions has not been reported in animals harbouring mutations in other Nucleoporins. However, it has been shown that localization of a Lamin, Otefin (Ote), to the nuclear envelope of GSCs is required for stem cell maintenance in female flies. Ote physically interacts with Medea to silence Bam in the GSCs. Over-expression of *ote* in the germ line cells increased the number of GSCs, implying that it is instructive for stem cell identity [30]. In contrast, *bam* expression at the tip of *nup98-96^2288^/Df(3R)mbc-R1* germaria did not extend into the GSC position, and overexpression of *nup98-96* had no effect on GSC number. These findings argue against the view that Nup98-96 acts in a common pathway with Ote. With all of the above observations taken together, it seems unlikely that the defects in maintaining early stage germ line cells in the *nup98-96^2288^/Df(3R)mbc-R1* mutants is due to a generic defect in nuclear pore or nuclear envelope structure, or general nucleocytoplasmic transport. Instead, it is likely that *nup98-96* function plays a specific role in the developmental timing between amplification and differentiation.

Recent studies have implicated the *nup98-96* gene products in a variety of specific functions in metazoans that appear to go beyond its role at the nuclear pore: *Arabidopsis thaliana* Nup96 was found to be required for basal immune-responses and constitutive resistance to non-host pathogens [31]; mouse Nup96 regulates the nuclear export of Interferon regulated mRNAs in immune responses [32]; and, finally, *Drosophila* Nup98 mediates gene transcription in response to the molting hormone Ecdysone [8], [9]. By analogy, we propose that the *nup98-96^2288^* allele eliminates a specific aspect of *nup98-96* function that is required for maintaining early stage germ line cells in an undifferentiated state. On a mechanistic level, this function could be mediated by transcriptional regulation or selective nucleocytoplasmic transport of factors required for timing the transition between amplification and terminal differentiation. While no such timing mechanisms have been identified in *Drosophila*, nuclear exclusion of the transcription factor Oct4, a master regulator of differentiation, is a prerequisite for maintaining mammalian tissue stem cells in an undifferentiated state [33].

In support of this view, the germ line cells in the *nup98-*96^2288^/Df*(3R)mbc-R1* mutant gonads were not responsive to the external cues that tightly control and influence the early stage germ line cells. EGFR-dependent signalling from the somatic support cells to the germ line cells promotes differentiation whereas activation of the TGF-β and JaK/STAT pathways promotes proliferation. A genetic analysis revealed that these pathways were not able to modify the number of amplification divisions in *nup98-96^2288^* gonads. *nup98-96^2288^* suppressed the effect of loss-of-function mutations in the EGFR pathway, which normally lead to dramatic over-proliferation of early germ line cells. Similarly, over-expression of TGF-β or JaK/STAT ligands in the germ line of *nup98-96^2288^/Df(3R)mbc-R1* animals remained without effect on germ line cell amplification. Furthermore, the expression pattern of *bam*, the main target of TGF-β signalling in the ovary, appeared normal in *nup98-*96^2288^/Df*(3R)mbc-R1* ovaries, suggesting that TGF-β signals act independently of *nup98-96*.

As targeted expression of a wildtype *nup98-96* cDNA in the germ line cells rescued the gonadal defects in *nup98-96^2288^* mutants, the function of Nup98-96 in maintaining an undifferentiated state reflects a germ line-intrinsic mechanism. Instead of regulating differentiation factors, *nup98-96* could be required cell-intrinsically for germ cell proliferation and a failure of the germ line cells to proliferate could trigger a cell-intrinsic differentiation response. The *nup98-96* locus in mice and *Drosophila* has been implicated in regulating proliferation. T-cells from Nup96+/− mice hyper-proliferate [9], [34], and overexpression of Nup98 in *Drosophila* embryonic S2-cells results in increased expression levels of cell cycle genes [9]. In contrast, the germ line cells in *nup98-96^2288^/Df(3R)mbc-R1* testes displayed an opposite response: reduced proliferation upon the loss of *nup98-96*. Overexpression of *nup98-96* in germ line cells, by way of UAST-*nup98-96,* did not increase the number of germ line cells in gonads from otherwise wildtype animals. These results demonstrate that the *Drosophila nup98-96* locus regulates a distinct response from the *nup98-96* locus in mouse.

The germ cell phenotype of *nup98-*96^2288^/Df*(3R)mbc-R1* animals adds to the current view of germ cell development and possibly to the development of other specialized cells from precursors. In testes but not ovaries, the *nup98-*96^2288^/Df*(3R)mbc-R1* mutant phenotype shares similarity to the *bam* mutant phenotype. Lack of *bam* has a different effect on the germ line cells in the two genders. In *bam* mutant ovaries, the germ line cells do not enter amplification divisions and accumulate as single cells. In testes, Bam regulates the number of mitotic amplification divisions. Reduction in *bam* expression causes the proliferation of spermatogonia beyond the 16-cell stage [35] and over-expression of Bam causes premature differentiation at the 8-cell stage [36]. It is possible that Bam is mis-expressed in the *nup98-*96^2288^/Df*(3R)mbc-R1* mutant testes and that this mis-expression contributes to the *nup98-*96^2288^/Df*(3R)mbc-R1* mutant phenotype. However, our observations emphasize that the *nup98-*96^2288^/Df*(3R)mbc-R1* mutant phenotype is different from the *bam* mutant phenotype. In *nup98-*96^2288^/Df*(3R)mbc-R1* mutant ovaries and testes, GSCs, their immediate daughters, and their mitotic progeny proliferated less than control cells and instead entered the differentiation program. Our findings suggest that the switch between GSC divisions, transit amplification divisions, and terminal differentiation has to be controlled at multiple levels. Further understanding of *nup98-96* function in the germ line cells awaits the identification of factors that regulate entry and exit from transit amplification divisions, and initiation of the terminal differentiation program.

## Materials and Methods

### Fly strains

Flies were raised on standard cornmeal molasses agar medium. The *nup98-96^2288^* mutation was identified by Antoine Guichet and Anne Ephrussi in a screen for flies with small gonads. Flies expressing RNAi-constructs for *nup98-96* (UAS-*nup98-96^RNAi^*, lines 31198 and 31199) were obtained from the Vienna *Drosophila* Resource Center. The *stet^1^* allele is described in [24]. *Df(3R)mbc-R1*, *nup98-96^339^*, UAS-*dpp*, UAS-*upd*, *nos-gal4:VP-16, C784*-*gal4*, *w^1118^*, Oregon R, balancer chromosomes, the 3^rd^ chromosome Deficiency kit, and mutants that mapped to the chromosomal area 95A-C are as described in [19] and were obtained from the Bloomington stock center.

### Mapping

The *nup98-96^2288^* mutation was mapped using Deficiencies spanning the 3^rd^ chromosome. Deficiencies *Df(3R)mbcR1* (95A5-7 to 95D6-11) and *Df(3R)mbc-30* (95A5-7 to 95D10-11) produced a germ line cell loss phenotype when in trans to *nup98-96^2288^* whereas deletions surrounding the area did not. Fly stocks carrying mutations in genes mapping to the 95A–C chromosomal region were tested for complementation. The *nup98-96^339^* mutation failed to complement *nup98-96^2288^* while all other mutants in the area complemented *nup98-96^2288^*.

### Molecular techniques

Generation of genomic DNA, sequencing, SDS-page, and Western blotting were performed following standard procedures [37]. Protein extracts were made from whole 1^st^ instar larvae. For western blots, chicken anti-Nup96-serum was used at 1∶20,000, rabbit anti-Nup98-serum was used at 1∶5,000, and mouse-anti-LaminC was used at 1∶70. Horseradish peroxidase coupled secondary antibodies for Western blotting were obtained from Promega (anti-chicken, 1∶5,000) and GE-Healthcare (anti-rabbit, 1∶20,000, anti-mouse 1∶10,000).

### Generation of UAST-*nup98-96*-constructs

Testis cDNA clones for *nup98-96* were obtained from the *Drosophila* Genomics Resource Center. The clone AT20377 contained 380 base pairs of the *nup98-96* 5′ prime sequence, which contains a TATA box for polymerase binding and the coding region for *nup98* (nucleotides 1 to 2250). The clone AT01311 contained the coding region for *nup96* and 500 base pairs of the *nup98-96* 3′ prime sequence, which contains consensus sequences for polyadenylation to assure transcript stability (nucleotides 2496 to 6522). A full-length clone of *nup98-96* was generated by standard molecular cloning techniques. A 1 kb fragment spanning the end of *nup98*, the auto-cleavage site, and the beginning of *nup96* was generated by PCR from genomic wildtype DNA and cloned directionally into the EcoR1 and EcoN1 restriction sites of AT01311, resulting in plasmid POT-CS-96. Subsequently, the *nup98-96* 5′ prime sequence and *nup98*-coding region were directionally cloned using EcoR1 and BspH1 into POT-CS-96 resulting in POT-*nup98-96*. The cDNA was cloned into a UAST-vector to generate UAST-*nup98-96*. To generate a *nup98*-cDNA, UAST-*nup98-96* was cut with Mlu1 and Nco1 to remove the *nup96* coding sequences, the ends were filled by the Klenow enzyme, and the vector was re-ligated. This resulted in a *nup98*-construct (UAST-*nup98*) that contains the *nup98-96* 5′ prime sequence, the nup98 coding region, the auto-cleavage site, a STOP codon, and the *nup98-96* 3′ prime sequence. The constructs were injected into flies by The Best Gene, Inc. Fly stocks were established from the injected animals and several independent lines were used for our experiments. All lines yielded the same results.

### UAS-*Gal4* expression studies

All crosses for cell-type specific expression (using the germ line cell nos*-gal4:VP-16* and the somatic cell *C784*-*gal4* transgene drivers) were set up and the subsequent F_1_ progeny raised at 29°C.

### Immunofluorescence and histochemistry

Tissues were dissected in testis buffer (10 mM Tris-HCl, ph 6.8, 180 mM KCl). Immunofluorescence was performed following standard procedures [38]. Tissues were observed using a Zeiss Axiophot microscope in brightfield and antibody (1∶2,000) was kindly provided by Dennis McKearin. Fluorescence-coupled secondary antibodies (Molecular Probes) were used at 1∶1,000. Tissues were embedded in Vectashield (Vector Laboratories) either with or without DAPI, or Slow Fade Gold (Molecular Probes).

### *In situ* hybridization

*In situ* hybridization was performed as previously described [22]. A full-length *piwi*-DNA in a pBST-vector for generation of the RNA-probes using the SP6 and T7 polymerase start sites was kindly provided by Dan Cox. fluorescent microscopy. Images were taken with a CCD camera using an Apotome and Axiovision Rel Software. Proteins for the production of polyclonal rabbit-anti-Nup98 (1∶500) and chicken-anti-NUP96 (1∶5000) were generated and purified by Enzymax. The anti-Nup98 antibody was raised against the 200 amino acids at the N-terminus of Nup98. The anti-Nup96 was raised against the 100 amino acids at the C-terminus of Nup96. Anti-Nup98 and anti-Nup96 antibodies were produced in and isolated from animals by Alpha Diagnostic. The following hybridoma/monoclonal antibodies were obtained from the Developmental Studies Hybridoma Bank, developed under the auspices of the NICHD, and maintained by The University of Iowa, Department of Biological Sciences, Iowa City, IA 52242: mouse anti-α-spectrin 3A9 (1∶10) developed by D. Branton and R. Dubreuil; mouse anti-LaminC (1∶10) developed by P. A. Fisher; mouse anti-Sex lethal M18 (1∶10) developed by P. Schedl; mouse anti-Groucho (1∶5) developed by C. Delidakis; and mouse anti-FasciclinIII 7G10 (1∶10) developed by C. Goodman. Goat anti-Vasa (1∶1000) and mouse anti-phosphorylated Jun-Kinase (1∶50) were obtained from Santa Cruz Biotechnology. Covance supplied the mouse anti-mAB414. Rabbit anti-phosphorylated Histone-H3 (1∶500) and mouse anti-Bromodioxyuridine (1∶200) were obtained from Millipore. Mouse-anti-Bam.

## References

[1] Gilbert SF (2006). Developmental Biology..

[2] Loeffler M, Potten CS (1997). Stem cells and cellular pedigrees – a conceptual introduction. In: Stem cells. Potten CS, editor.. Elsevier Academic Press.

[3] Jones DL, Yamashita Y, Schulz C, Fuller MT, Lanza R, Gearhart J, Hogan B, McKay R, Melton D, Pedersen R, Thomson J, West M (2004). Regulation of stem cell self-renewal versus differentiation by a stem cell niche: Lessons from the *Drosophila* male germ line.. Handbook of stem cells.

[4] Gilboa L, Lehmann R (2004). How different is Venus from Mars? The genetics of germ-line stem cells in *Drosophila* females and males.. Development.

[5] Adam SA (2001). The nuclear pore complex.. Genome Biol.

[6] Rout MP, Aitchison JD (2001). The Nuclear Pore Complex as a Transport Machine.. J Biol. Chem.

[7] Suntharalingham M, Wente SR (2003). Peering through the Pore: Nuclear Pore Complex Structure, Assembly, and Function. Dev.. Cell.

[8] Capelson M, Liang Y, Schulte R, Mair W, Wagner U (2010). Chromatin-bound Nuclear Pore Components Regulate Gene Expression in Higher Eukaryotes.. Cell.

[9] Kalverda B, Pickersgill H, Shloma VV, Fornerod M (2010). Nucleoporins Directly Stimulate Expression of Developmental and Cell-Cycle Genes Inside the Nucleoplasm.. Cell.

[10] Wente SR, Blobel G (1994). Nup145 encodes a novel yeast glycine-leucine-phenylalanine-glycine (GLFG) nucleoporin required for nuclear envelope structure.. J Cell Biol.

[11] Fontoura BMA, Blobel G, Matunis MJ (1999). A Conserved Biogenesis Pathway for Nucleoporins: Proteolytic Processing of a 186-Kilodalton Precursor Generates Nup98 and the Novel Nuleoporin, Nup96.. J Cell Biol.

[12] Presgraves DC, Balagopolan L, Abmayr SM, Orr HA (2003). Adaptive evolution drives divergence of a hybrid inviability gene between two species of *Drosophila*.. Nature.

[13] Hardy RW, Tokuyasu KT, Lindsley DL, Garavito M (1979). The germinal proliferation center in the testis of *Drosophila melanogaster*.. J of Ultrastructural Res.

[14] Fuller MT, Bate M, Martinez AriasA (1993). Spermatogenesis in *Drosophila*. The Development of Drosophila melanogaster.

[15] Matunis E, Tran J, Gönczy P, DiNardo S (1997). *punt* and *schnurri* regulate a somatically derived signal that restricts proliferation of committed progenitors in the germ line.. Development.

[16] Kiger AA, White-Cooper H, Fuller MT (2000). Somatic support cells restrict germ line stem cell self-renewal and promote differentiation.. Nature.

[17] Lin H, Yue L, Spradling AC (1994). The *Drosophila* fusome, a germ line-specific organelle, contains membrane skeletal proteins and functions in cyst formation.. Development.

[18] Spradling AC, Bate M, Martinez AriasA (1993). Oogenesis.. The Development of Drosophila melanogaster.

[19] The Flybase Consortium (2003). The FlyBase database of the *Drosophila* genome projects and community literature.. Nucleic Acids Res.

[20] Phelps CB, Brand AH (1998). Ectopic gene expression in *Drosophila* using GAL4 system.. Methods.

[21] Van Doren M, Williamson AL, Lehmann R (1998). Regulation of zygotic gene expression in *Drosophila* primordial germ line cells.. Curr. Biol.

[22] Ohlstein B, McKearin DM (1997). Ectopic expression of the *Drosophila* Bam protein eliminates oogenic germ line stem cells.. Development.

[23] Song X, Wong MD, Kawase E, Xi R, Ding BC (2004). Bmp signals from niche cells directly repress transcription of a differentiation-promoting gene, *bag of marbles*, in germ line stem cells in the *Drosophila* ovary.. Development.

[24] Schulz C, Kiger AA, Tazuke SI, Yamashta YM, Pantalena-Filho LC (2004). A mis-expression screen reveals effects of *bag-of-marbles* and TGFβ class signalling on the *Drosophila* male germ line stem cell lineage.. Genetics.

[25] Bunt SM, Hime GR (2004). Ectopic Activation of Dpp Signalling in the Male *Drosophila* Germ Line Inhibits Germ Line Cell Differentiation.. Genesis.

[26] Schulz C, Wood CG, Jones DL, Tazuke SI, Fuller MT (2002). Signalling from germ line cells mediated by the rhomboid homologue *stet* organizes encapsulation by somatic support cells.. Development.

[27] Sarkar A, Parikh N, Hearn SA, Fuller MT, Tazuke SI (2007). Antagonistic roles of Rac and Rho in organizing the germ cell micro-environment.. Curr. Biol.

[28] Kiger AA, Jones DL, Schulz C, Rogers MB, Fuller MT (2001). Stem cell self-renewal specified by JAK-STAT activation in response to a support cell cue.. Science.

[29] Tulina M, Matunis E (2001). Control of stem cell self-renewal in *Drosophila* spermatogenesis by JAK-STAT signalling.. Science.

[30] Jiang X, Xia L, Chen D, Yang Y, Huang H (2008). Otefin, a Nuclear Membrane Protein, Determines the Fate of Germ line Stem Cells in *Drosophila* via Interaction with Smad Complexes.. Dev.Cell.

[31] Zhang Y, Li X (2005). A putative nucleoporin 96 is required for both basal defense and constitutive resistance responses mediated by suppressor of npr1-1, constitutive 1.. Plant Cell.

[32] Faria AMC, Levay A, Wang Y, Kamphorst AO, Rosa MLP (2006). The Nucleoporin Nup96 Is Required for Proper Expression of Interferon-Regulated Proteins and Functions.. Immunity.

[33] Pan G, Qin B, Liu N, Schoeler H, Pei D (2004). Identification of a Nuclear Localization Signal in OCT4 and Generation of a Dominant Negative Mutant by Its Ablation.. J Biol. Chem.

[34] Chakraborty P, Wang Y, Wei JH, van Deursen J, Yu H (2008). Nucleoporin levels regulate cell cycle progression and phase-specific gene expression.. Dev.Cell.

[35] McKearin DM, Spradling C (1990). Bag-of-marbles: A Drosophila gene required to initiate both male and female gametogenesis.. Genes dev.

[36] Insco ML, Leon A, Tam CH, McKearin DM, Fuller MT (2009). Accumulation of a differentiation regulator specifies transit amplifying division number in an adult stem cell lineage.. PNAS.

[37] Sambrook J, Fritsch EF, Maniatis T (1989). Molecular cloning..

[38] Ashburner M (1989). Drosophila..

